# A personalised prosthetic liner with embedded sensor technology: a case study

**DOI:** 10.1186/s12938-020-00814-y

**Published:** 2020-09-14

**Authors:** Linda Paternò, Vimal Dhokia, Arianna Menciassi, James Bilzon, Elena Seminati

**Affiliations:** 1 Department of Mechanical Engineering, University of Bath, Bath, UK; 2 The BioRobotics Institute, Scuola Superiore Sant’Anna, Pisa, Italy; 3 Department for Health, University of Bath, Bath, UK; 4 CAMERA Centre, University of Bath, Bath, UK

**Keywords:** Cryogenic CNC machining, Temperature, Humidity, Lower limb, Prosthetic liner, Prosthetic socket, Transtibial amputation

## Abstract

**Background:**

Numerous sensing techniques have been investigated in an effort to monitor the main parameters influencing the residual limb/prosthesis interface, fundamental to the optimum design of prosthetic socket solutions. Sensing integration within sockets is notoriously complex and can cause user discomfort. A personalised prosthetic liner with embedded sensors could offer a solution. However, to allow for a functional and comfortable instrumented liner, highly customised designs are needed. The aim of this paper is to presents a novel approach to manufacture fully personalised liners using scanned three-dimensional image data of the patient’s residual limb, combined with designs that allow for sensor integration. To demonstrate the feasibility of the proposed approach, a personalised liner with embedded temperature and humidity sensors was realised and tested on a transtibial amputee, presented here as a case study.

**Methods:**

The residual limb of a below knee amputee was first scanned and a three-dimensional digital image created. The output was used to produce a personalised prosthesis. The liner was manufactured using a cryogenic Computer Numeric Control (CNC) machining approach. This method enables fast, direct and precise manufacture of soft elastomer products. Twelve Hygrochron Data Loggers, able to measure both temperature and humidity, were embedded in specific liner locations, ensuring direct sensor-skin contact. The sensor locations were machined directly into the liner, during the manufacturing process. The sensors outputs were assessed on the below amputee who took part in the study, during resting (50 min) and walking activities (30 min). To better describe the relative thermal properties of new liner, the same tests were repeated with the amputee wearing his existing liner. Quantitative comparisons of the thermal properties of the new liner solution with that currently used in clinical practice are, therefore, reported.

**Results:**

The liner machining process took approximately 4 h. Fifteen minutes after donning the prosthesis, the skin temperature reached a plateau. Physical activity rapidly increased residuum skin temperatures, while cessation of activity caused a moderate decrease. Humidity increased throughout the observation period. In addition, the new liner showed better thermal properties with respect to the current liner solution (4% reduction in skin temperature).

**Conclusions:**

This work describes a personalised liner solution, with embedded temperature and humidity sensors, developed through an innovative approach. This new method allows for a range of sensors to be smoothly embedded into a liner, which is capable of measuring changes in intra-socket microclimate conditions, resulting in the design of advanced socket solutions personalised specifically for individual requirements. In future, this method will not only provide a personalised liner but will also enable dynamic assessment of how a residual limb behaves within the socket during daily activities.

## Background

The interaction between the residual limb and the socket (*i.e.* the physical human–machine interface of limb prostheses) is of paramount importance to ensure an efficient fitting of the prosthesis that provides stability, mobility control and comfort for the amputee [1]. To keep the prosthetic limb attached to the body, different suspension systems can be selected. Often, the suspension includes a prosthetic liner, *i.e.* a soft cover which enhances comfort and protects the tissues of the residual limb not accustomed to bearing loads [2].

Generally, transtibial sockets are personalised handmade carbon fiber-laminated sockets which can be classified into, (1) Specific Surface Bearing sockets, such as the Patellar Tendon Bearing (PTB) or the Patellar Tendon Kegel (PTK) sockets, and, (2) Total Surface Bearing sockets. The first class includes socket designs characterised by a proximal “brim” shape which creates a supracondylar grip on the bony prominences, increasing the prosthesis stability. The second class aims to equally distribute the load on the total socket surface to avoid high stresses on biological tissues.

Liners can be made of elastomeric materials (gels or silicones) or of open/closed cell foam materials. Elastomeric liners are rolled on the residual limb ensuring stability as a result of the material friction and the created suction effect [2–5]. These roll-on liners can be locked to the socket by a distal mechanism (*e.g.* pin lock, magnetic lock, lanyard strap) or by a sub-atmospheric suspension created through a passive unidirectional valve or through an active vacuum pump. By measuring the residual limb circumference at different levels, a prosthetist can identify the most appropriate size of a commercial liner [6]. Different liner types can be selected by the prosthetist mainly by referring to the product literature and prior personal experience. Depending on the residual limb features, commercial liners can be made of different elastomeric materials and with different thicknesses and profiles (cylindrical or conical), but they are not typically personalised to meet the patient’s residuum requirements. However, for some specific cases where the shape of the residuum is compromised and/or complex (*e.g.* following traumatic amputation), personalised handmade liners are inevitable [7]. Open or closed cell foam liners, such as Pe-Lite liners, are another popular solution and are typically manufactured by the prosthetist. These liners are coupled with a neoprene or latex suspension sleeve worn over the socket extending to the thigh region [8]. In general, since residual limbs change in shape and volume over time, socks are commonly worn between the residual limb and the liner to provide a degree of volume and shape compensation. This avoids the need for multiple new prosthetic sockets, that are expensive and time-consuming to manufacture. The use of socks offers a simple and low-cost solution. However, the risk of localized stresses and skin tensions increases and, as a consequence, the risk of dermatological problems (*e.g.* blisters and skin abrasions). In addition, this can be very uncomfortable for patients who have to wear several socks to compensate volume fluctuations, making the prosthesis donning and doffing a time-consuming activity.

Previous literature on transtibial sockets and liners is difficult to interpret because of the wide range of factors that play a key role at the residual limb/prosthesis interface. However, it is clear that there is a need to improve the present socket system solutions to overcome the dermatological problems and discomforts presented by lower limb amputees [2, 6, 9]. The combination of the liner/socket shape and material are key determinants of the stresses acting on the residuum tissues. High pressures and shear stresses can generate skin breakdowns, irritations and associated pain. In addition, the liner, together with the socket, creates a physical barrier to the thermal transfer mechanisms of the skin, thus compromising the thermal homeostasis of the residual limb within the prosthesis [10]. Excessive sweating can cause the prosthesis to slip off. This affects the suspension and the fitting of the prosthesis, increasing the random movement of the residual limb in relation to the socket. The sweat makes the skin softer and more prone to blister formation or skin maceration. Moreover, altering the stress distribution and the thermal homeostasis at the residual limb/socket interface can increase the volume fluctuations of the residual limb. Monitoring and comparing these parameters in different prosthetic socket and liner solutions is the first step towards the design of advanced systems able to improve the patient’s comfort and quality of life. Therefore, in the past, several studies have been dedicated to the quantitative assessment of the different parameters affecting this interface.

**Fig. 1:**
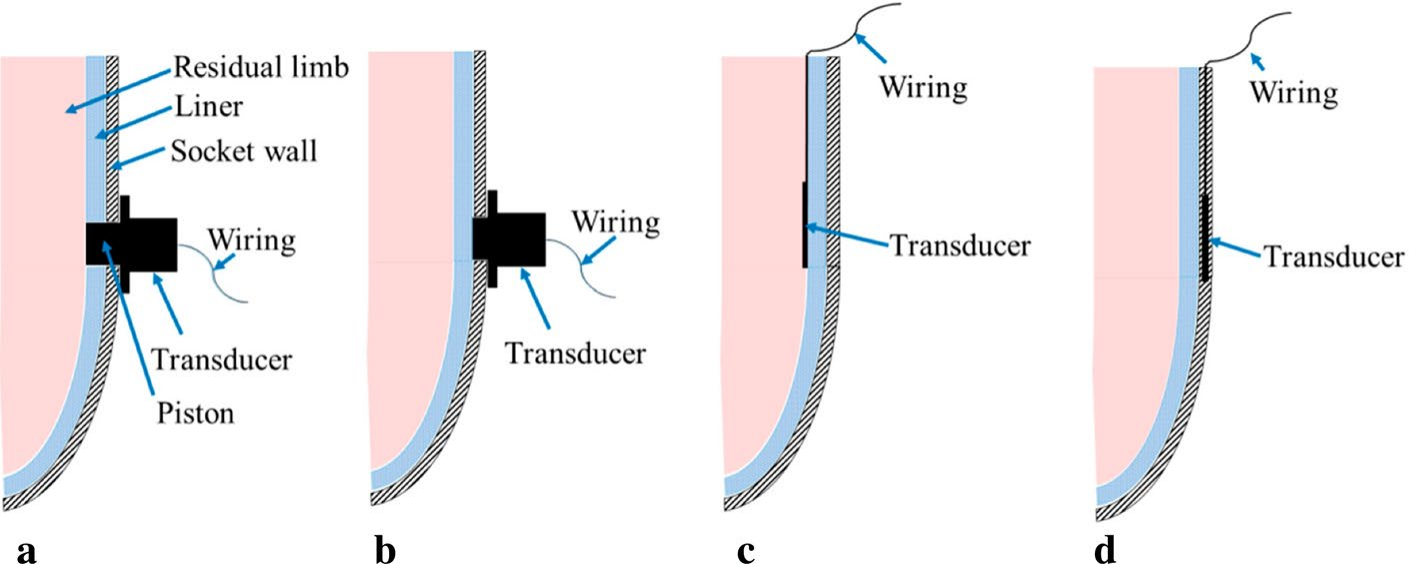
Sensing systems mounting techniques for interface measurements within transtibial prosthetic socket: integrating sensors on the socket wall (**a**) in contact with the skin or (**b**) with the prosthetic liner; (**c**) inserting sensors within the socket; (**d**) embedding the sensors into the socket wall (adapted by [11])

Various sensing systems have been applied for interfacial measurements within transtibial prosthetic socket [9]. However, several issues are reported in the literature, mainly related to the sensor mounting technique at the residual limb/socket interface. The challenge is to ensure that sensor integration does not impair the prosthesis fitting, while still ensuring a direct sensor-skin contact. Three contemporary approaches have been used [11]: integrating sensors on the socket wall (Fig. 1a, b), inserting sensors within the socket (Fig. 1c), and embedding sensors into the socket wall (Fig. 1d).

Inserting sensors within the socket restricts the available options, requiring extra thin yet durable technology. This often results in poor sensing properties in terms of key parameters, including accuracy and sensitivity. On the contrary, the integration on and into the socket wall has the main disadvantage of requiring an ad hoc socket design, which involves a considerable amount of skilled prosthetist manual work, which is expensive and time consuming [12]. Reverse engineering, computer aided design (CAD/CAM) and 3D printing techniques are beginning to be applied in the design and manufacturing of prosthetic sockets and could overcome these limitations [13–18]. However, further research is still required to improve the structural integrity of 3D printed sockets.

An alternative solution is the design and manufacture of an ad hoc prosthetic liner which allow for a smooth sensor integration at the skin interface without socket modifications. A sensorised liner should be designed, personalised to the patient’s requirements to avoid discomfort and manufactured in a cost-effective way. Nevertheless, to date, personalised liners are still manufactured by prosthetists using traditional handmade methods resulting in a labour-intensive process. Plaster casts are wrapped around the residual limb to capture the shape and create the negative cast [7, 19]. The positive cast is obtained from the negative and rectifications are made by the prosthetist to obtain the final shape of the liner. Subsequently, the liner is thermoformed on the positive cast using either open or closed cell foams. For elastomeric liners, a thickness is created on the positive cast (*e.g.* by applying layers of cork) and a mould is thermoformed on it [7]. The layers are then removed, and the elastomeric liner is manufactured using injection moulding techniques between the cast and the mould itself.

There is a need for new solutions for designing and manufacturing personalised liners which can easily integrate sensor technologies. This will allow a biomechanical/physiological characterization of the prosthesis interface and enable objective comparisons and indicators of socket system quality. Currently, 3D printing methods for soft materials are too expensive for prosthetic liners, characterised by long manufacturing times, poor scalability and material properties that exhibit poor fatigue qualities for this purpose [20, 21]. Therefore, other techniques need to be investigated. Cryogenic Computer Numeric Control (CNC) machining for soft polymer is a potential method for rapidly manufacturing soft material products [22]. This process is based on directly freezing a billet of elastomeric material to below its glass transition temperature (*T*_*g*_) and then directly machine it using traditional CNC machine tools. This results in a fast and cost-effective manufacturing solution that can generate prototypes with a high degree of accuracy and conformity. Previous studies have already demonstrated that this technique could be applied to the manufacture of personalised symptom-specific sports insoles starting from the scan of the subject’s feet [23] and prosthetic liner components starting from the scan of residual limb models [22].

This paper presents one of the first attempts to develop a novel fully personalised liner with embedded sensors via a semi-automated process. The purpose of this liner is to provide precise fitting and monitoring of key residual limb/prosthesis interface parameters. To demonstrate the feasibility of the novel approach and to evaluate the new liner outcomes, humidity and temperature sensors (*i.e.* Hygrochron Data Loggers iButtons) were embedded into the liner, with the possibility to embed other sensors in the future. The design of the liner was based on the characteristics of the residuum of a unilateral below the knee amputee, as a case study example. The design and the manufacturing of the prototype are described along with a set of experimental tests where the recruited participant wore the new liner while performing typical daily activities. The same set of experimental tests was also repeated with the patient’s current prosthetic liner to provide a comparative assessment.

## Results

### Skin sensor calibration

**Fig. 2:**
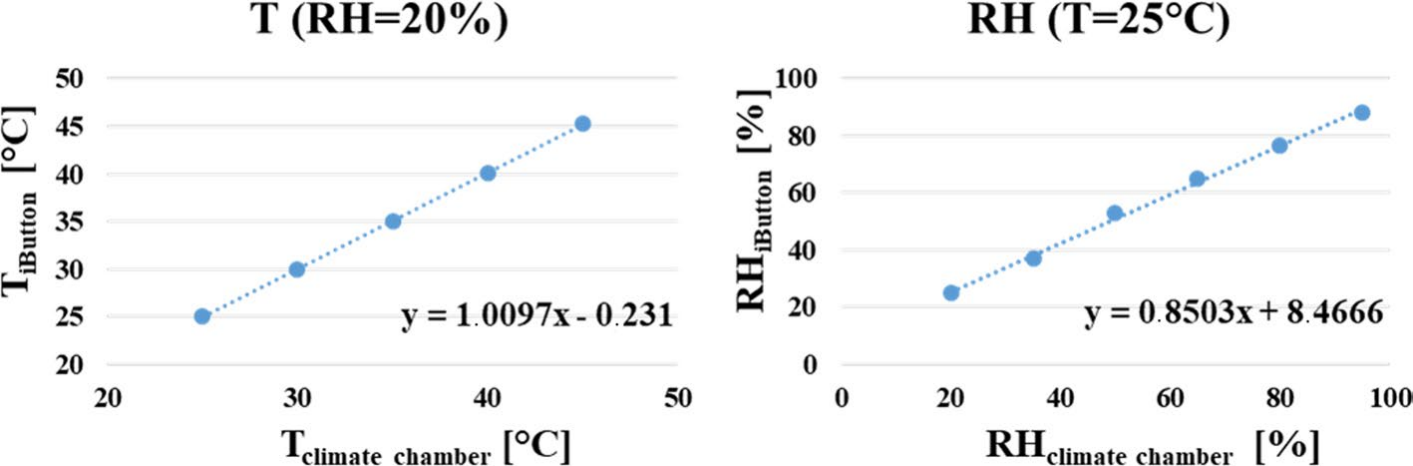
Example of the linear regression for the temperature (T) (left) and the relative humidity (RH) (right) measured by one iButton and the climate chamber during the calibration process. Considering all the iButtons, the linear regression resulted in a mean slope coefficient equal to 1.00 ± 0.01 for the temperature (range: 0.89–1.07) and to 0.88 ± 0.013 for the relative humidity (range: 0.83–0.91). The mean intercept was 0.36 ± 0.11 for the temperature (range: − 1.84 to 4.87) and 6.98 ± 0.51 for the relative humidity (range: 5.70–9.12)

Twelve Hygrochron Data Loggers iButtons were calibrated and subsequently embedded in the new personalised liner. Data measured by the sensors were compared and numerical values derived from regression relationships recorded in a controlled climatic chamber. Linear regression lines were constructed for both temperature and relative humidity for each iButton (an example is reported in Fig. 2). The correlation coefficients (*i.e.* the measurement of the linear correlation between the two variables T_iButton_/T_climate chamber_ and RH_iButton_/RH_climate chamber_–details in Fig. 2) were derived for each temperature/relative humidity condition and for each iButton, resulting in always being very close to 1 at less than 0.1 (≥ 0.99). This confirms a strong positive linear correlation between the temperature/relative humidity values measured by the iButton sensors and the climate chamber.

The mean accuracy and the mean precision for both temperature and relative humidity before and after applying the correction formula for each iButton sensor are reported in Table 1, confirming that the calibration process is required for each iButton prior to use in a research setting.

**Table 1:** Accuracy and precision before and after the calibration process of the iButtons

	Accuracy		Precision	
	T [°C]	RH [%]	T [°C]	RH [%]
Before calibration	0.53 ± 0.08 range: 0.40 to 0.65	− 0.16 ± 0.45 range: − 1.02 to 0.66	0.29 ± 0.02 range: 0.24 to 0.31	3.62 ± 0.39 range: 3.07 to 4.43
After calibration	0.05 ± 0.09 range: 0.03 to 0.07	0.06 ± 2.14 range: − 0.97 to 2.17	0.19 ± 0.31 range: 0.03 to 0.14	1.6 ± 0.76 range: 0.8 to 2.11

### Personalised and sensorised liner

**Fig. 3:**
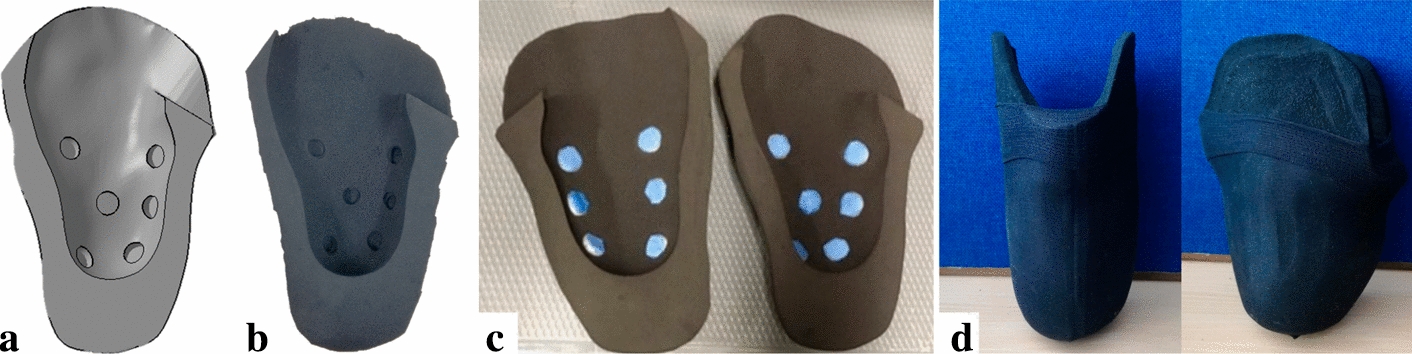
**a**, **b** CAD and Neoprene foam prototype of the new personalised liner with specific enclosures designed to embed the Hygrochron Data Loggers iButtons; **c** the two refined halves of the new personalised liner with the twelve embedded Hygrochron Data Loggers covered with the layer of breathable yet waterproof fabric; **d** the final personalised liner prototype

The final new fully personalised liner with specific blind enclosures to embed sensors at the skin interface is shown in Fig. 3. The entire manufacturing process, using the cryogenic CNC machining approach lasted approximately 4 h and resulted in a design that matched the residuum shape of the amputee patient. The liner weight was measured as 107.3 g, resulting in a lighter solution compared with the state-of-the-art solutions. Indeed, the average weight of transtibial prosthetic liners is approximately 450 g, which is approximately 30% of the total prosthesis weight [24]. For example, the patient’s Pe-lite liner system weighted 469.2 g (63.4 g × 4 of the socks + 107.4 g of the Pe-Lite Liner + 108.2 g of the SILOSHEATH™ prosthetic sheath).

### Human participant tests

**Fig. 4:**
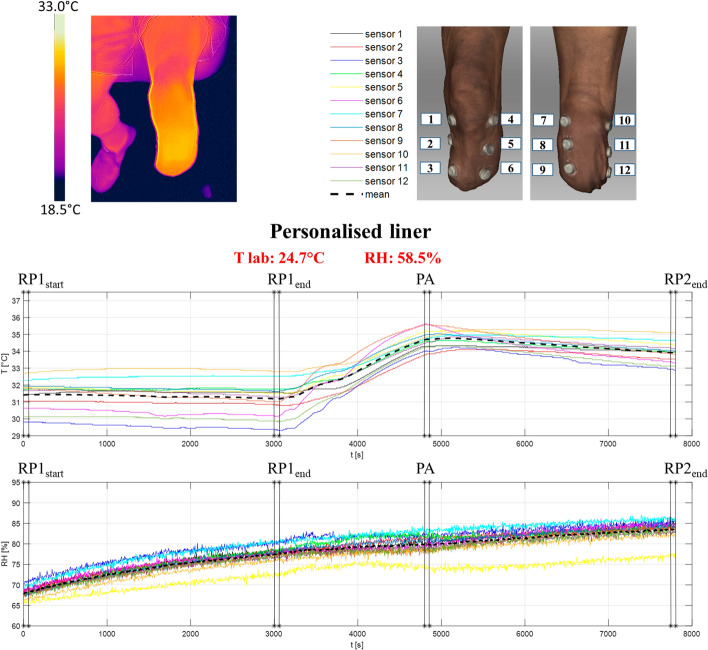
Left top corner: Test image acquired by the thermo-camera at the end of the test session (the image has been recorded in the frontal view at the end of the protocol). Right top corner: iButtons positions on the residual limb during the test session (anterior and posterior views). Bottom: Temperature and relative humidity values within the prosthetic socket with the new liner. The vertical lines indicate the different 1-min stages used to analyse the data: RP1_start_, first minute of the 1st resting period (test minute 1); RP1_end_, last minute of the 1st resting period (test minute 50); PA, last minute of walking on the treadmill (test minute 80); RP2_end_, last minute of the 2nd resting period (test minute 130). Temperature and humidity room conditions are reported in red on the top of the graphs

The skin temperature and intra-socket relative humidity recorded with the new liner during the entire data collection periods (50-min seated resting period, 30-min of walking at self-selected speed on a treadmill and 50-min seated resting period post-exercise) are shown in Fig. 4. Different stages of the tests are identified:

1. RP1_start_: start of the 1st resting period (first minute of the 1st resting period; test minute 1);
2. RP1_end_: end of the 1st resting period (last minute of the 1st resting period; test minute 50);
3. PA_end_: end of the physical activity (last minute of walking on the treadmill; test minute 80);
4. RP2_end_: end of the 2nd resting period (last minute of the 2nd resting period; test minute 130).

At the beginning of the tests (RP1_start_), the skin temperature and relative humidity were 31.42 ± 0.86 °C and 68.13% ± 1.33%, respectively (average across the 12 sensors ± standard deviation) (Table 2). Approximately, 15-min after donning the prosthesis, the skin temperature plateaued at 31.20 ± 1.01 °C at the end of the first resting period (RP1_end_). Physical activity caused a rapid increment in skin temperature, which reached a mean value of 34.62 ± 0.57 °C (at PA_end_), while cessation of activity caused a moderate decrease with a final mean value of 33.91 ± 0.63 °C (at RP2_end_). This was qualitatively confirmed by the thermo-camera image at the end of the test session (see left top corner of Fig. 4).

**Table 2:** Temperature and relative humidity mean values ± standard deviation (std) of the residual limb skin when wearing the new personalised liner (average of the 12 sensors)

New personalised liner		
	T mean ± std [°C]	RH mean ± std [%]
RP1_start_	31.42 ± 0.86	68.13 ± 1.33
RP1_end_	31.20 ± 1.01	77.37 ± 2.11
PA_end_	34.62 ± 0.57	79.87 ± 2.39
RP2_end_	33.91 ± 0.63	83.48 ± 2.28

Data were averaged on the initial/final 1-min recorded data of each task (see in Fig. 4 for details)

On the contrary, intra-socket relative humidity continued to increase throughout the data collection period, with maximal values reached at the end of the protocol (77.37 ± 2.11% at RP1_end_, 79.87 ± 2.39% at PA_end_ and 83.48 ± 2.28% at RP2_end_.

The values recorded by each sensor during the different tasks are reported in Table 1 of the Additional file 1. Each value represents the mean value on the 1-min interval at the beginning and end of the task.

**Fig. 5:**
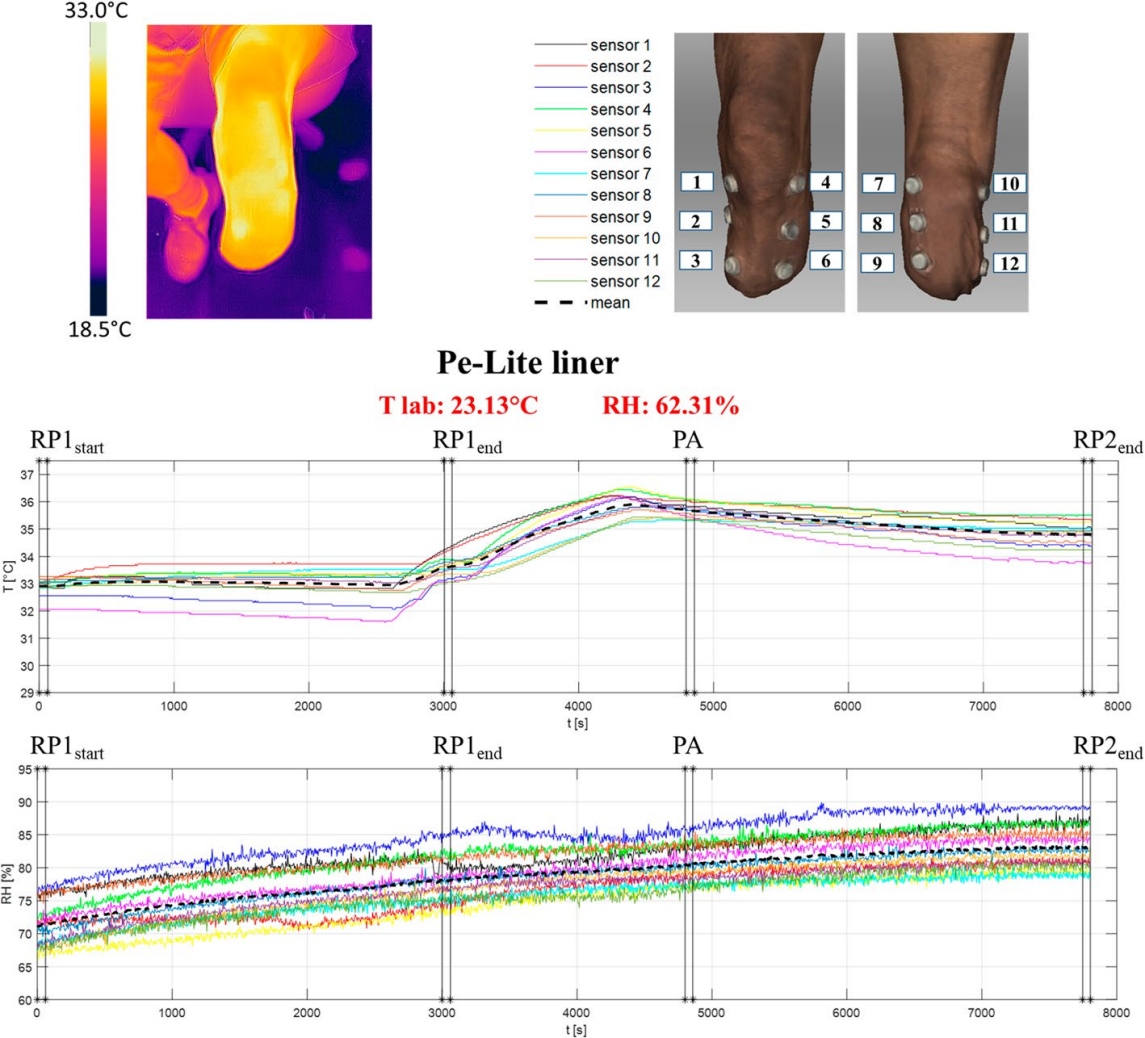
Left top corner: Test image acquired by the thermo-camera at the end of the test session with the patient’s Pe-Lite liner (the image has been recorded in the frontal view at the end of the protocol). Right top corner: iButtons positions on the residual limb during the test session (anterior and posterior views). Bottom: Temperature and relative humidity values within the prosthetic socket with the Pe-Lite liner. The vertical lines indicate the different 1-min stages used to analyse the data: RP1_start_, first minute of the 1st resting period (test minute 1); RP1_end_, last minute of the 1st resting period (test minute 50); PA, last minute of walking on the treadmill (test minute 80); RP2_end_, last minute of the 2nd resting period (test minute 130). Temperature and humidity room conditions are reported in red on the top of the graphs

To provide a relative comparison of the thermal responses to the new personalised solution, the same test protocol was repeated when the amputee participant was wearing his current liner. Results are showed in Fig. 5 and the mean values for the initial/final 1-min of each task are also reported in Table 3.

**Table 3:** Temperature and relative humidity mean values ± standard deviation (std) of the residual limb skin (average of the 12 sensors)

Pe-lite liner system		
	T mean ± std [°C]	RH mean ± std [%]
RP1_start_	32.91 ± 0.32^**^	71.25 ± 3.54^**^
RP1_end_	33.52 ± 0.38^**^	77.94 ± 3.48
PA_end_	35.72 ± 0.28^**^	80.37 ± 3.01
RP2_end_	34.80 ± 0.49^**^	83.07 ± 3.34

Data were averaged on the initial/final 1-min recorded data of each task (see in Fig. 4 for details)

**Indicate significant differences (*p* < 0.01) between the two liners systems after the paired T-test

There were significant differences in skin temperature between the two liner solutions at all four stages of the test, being lower in the new personalised instrumented liner solution. The new personalised liner also resulted in significantly lower measures of intra-socket humidity at the first stage (RP1_start_) of the protocol. The thermal properties of the new personalised liner appear to allow for a better heat dissipation and the maintenance of lower skin temperature over time (see Table 3 for details). The thermo-camera images acquired at the end of the test sessions qualitatively confirm that the temperature distribution after 130-min of wearing the prosthesis was lower with the new personalised liner (see left top corner of Fig. 5).

The results of the comfort analysis on the amputee participant showed that the new personalised liner scored 8/10 on the Socket Comfort Score (SCS) and was perceived equivalent in comfort to the standard provision. Qualitative feedbacks obtained with a modified Prosthesis Evaluation Questionnaire (PEQ) supported the use of the new solution, with no element rated below 67% of total satisfaction, thus verifying the acceptance of the new solution.

## Discussion

The new paradigm for the development of fully personalised prosthetic liners, reported in this paper could offer an efficient solution for the manufacture of ad hoc liners. The cryogenic CNC machining approach [22], coupled with a 3D scanning technique has shown to be a potential alternative to the traditional handmade method for prosthetic liner manufacture. The cryogenic CNC approach allows for low-cost personalised liner solutions within 4–6 h, considerably less than traditional methods that can take several weeks [3]. Beginning with the 3D scan of the patient’s residual limb, the personalised liner is designed. This liner is designed to precisely match the contours of the patient’s residual limb. This new type of liner provides an alternative solution for the interface of transtibial amputees with their prosthesis. These advantages can be extremely useful when the residuum is affected by large volume fluctuations. A set of liners of different sizes and shapes could be designed and manufactured offering an alternative to the use of several layers of socks. This can be critical during the early amputation stage (over a typical time period of 12–18 months after amputation), when the residuum volume is reducing rapidly. Furthermore, for children with an amputation, who are required to continually change the liner across their lifetime, as a result of volume and shape changes during growth and maturation. The manufacture of new prosthetic sockets is expensive and time consuming. Therefore, the proposed personalised liner approach could be an alternative solution to ensure a proper prosthesis fitting over a longer period of time.

The new liner is also lighter compared with other commercial liners/interface solutions currently available. In addition, through personalising the internal surface of the liner, sensors can be embedded into strategic locations. The data captured through the sensorised liner can be used to help design subsequent liners and sockets tuned for the wearer’s requirements. The present case study focused on temperature and relative humidity sensors, but this new solution could be used in future applications to embed different sensors that can quantitatively assess the residuum in a synergistic way (*e.g.* not only temperature and relative humidity but also pressure and shear stress, etc.) [25, 26]. This could mean specifically manufactured geometrical attributes within liner and socket to take account of bony landmarks or areas subjected to high pressures and efficient material selections for the thermal homeostasis of the limb. Through the constant monitoring of the residuum interface conditions, many issues related to the skin damages and pain could be avoided, thus improving the comfort and the hygiene of the residual limb within the prosthesis.

The comfort of the liner, assessed via the modified version of the PEQ and the SCS Socket Comfort Score, confirmed the acceptability of the proposed solution, together with the very positive feedback reported by the case study participant. These outcomes remain to be substantiated as part of a large clinical trial, but they reflect important features of the liner related to possible pain and comfort during daily life activities and, therefore, their impact on user comfort and quality of life.

With regards to the thermal environment characterization within a prosthetic socket, there are very few clinical studies that address this issue [12]. Klute et al. [27] measured skin temperature at 16 different sites using thermistor sensors on 9 unilateral transtibial amputees. The protocol included 60-min of sitting, 30-min of treadmill walking at a self-selected speed and 60-min of sitting. The results showed a mean temperature of 31.0 ± 1.5 °C after the initial 60-min sitting, then an increment of 3.1 °C after walking and, finally, a mean temperature of 33.2 ± 1.2 °C at the end of the test. The highest temperature was measured over the tibialis anterior area. In general, temperatures were reported to be higher at the proximal sites than at the distal sites and near muscular locations rather than bony ones. These results are similar to those reported in the previous clinical studies including case studies [28]. Also Peery et al. [29] analysed in-socket temperatures using thermistor sensors. The protocol included five transtibial amputees and measured the temperature at 14 locations during a seated resting period of 15-min and a walking period of 10 min. The initial mean skin temperature result was 31.4 ± 1.3 °C. Then, it increased by 0.8 °C at the end of the resting period and reached 33.1 ± 1.8 °C at the end of the test. In general, the skin was cooler in the areas of low perfusion (*e.g.* across anterior location) and warmer in the areas of high perfusion (*e.g.* across the posterior region). The results obtained in this study confirm the assumptions of the previous works (see Additional file 1). Indeed, in general, distal-anterior sensors (number 3 and 6 in Figs. 4 and 5) measured lower temperatures especially during the first 50-min resting period while the sensors number 7 and 8, near muscular locations, showed higher temperature values. Similarly, also in this study, the temperature increased by 2–3 °C after the 30-min of physical activity and it did not come back to the original values even after 50-min of rest (sitting on a chair).

Generally, more than 53% of prosthetic users feel discomfort due to excessive heat or sweating, and an increment of 1–2 °C is sufficient to cause this kind of problem [2]. Because amputees have less surface area to allow heat dissipation, the excessive perspiration negatively affects the residual limb/socket interfaces. For this reason, relative humidity is another important parameter that should be monitored at the residuum interface. Instability, skin maceration and bacterial invasion may occur especially in patients affected by vascular diseases [2]. These patients represent the vast majority of the amputees and they have compromised thermoregulatory responses (*e.g.* reduced capacity to vasoconstrict in response to cold environments and vasodilate in response to warm environments) [30]. In addition, when the skin is moist, the frictional load on the residuum will be higher, facilitating irritations and blisters, making it impossible for the amputee to wear the prosthesis.

This study is one of the first attempts to investigate the effect of physical activity on intra-socket relative humidity of a lower limb amputee. Indeed, the limitations due to the sensing mounting technique within the socket highly restrict the available sensors to be used in this type of clinical study. The majority of previous research reports only the temperature characterization obtained by thermistor sensors (smaller and thinner with respect to the Hygrochron Data Loggers iButtons selected in our study). The new approach described in this paper can help to overcome these limitations allowing for a wide range of sensors to be embedded.

In comparison to temperature results, relative humidity was characterised by a continuously increasing trend not only during the rest period and the physical activity, but also during the rest period after exercise (~ 3%). This suggests that the amputee needs to stop periodically to doff the prosthesis and remove the sweat. In addition, when donning the prosthesis, the new personalised liner showed lower values of relative humidity if compared with the usual patient’s Pe-Lite system. However, there was an increase of 7% and 9%, respectively, for the Pe-Lite and the new liner after 50-min of resting. The exercise (30-min treadmill walking) increased the relative humidity by 2–3% for both liner systems, showing similar results to a previous preliminary study [31] that measured an increase of 4% relative humidity in the socket after 15-min of walking activity. However, only one sensor was used, and the absolute values at resting were not reported.

Although the outcomes of this study are promising, there are some limitations. The current liner is made up of two different components, which requires considerable CAD expertise to create for the practitioners/prosthetists. However, this technique can significantly reduce the time required to prepare/adjust amputee sockets/liners components, avoiding the need for prosthetists to engage in labour-intensive processes to modify sockets using manual methods (using cast as a mould and plaster models). In the future, through the development of automated CAD scripts that is driven by key landmarks, the design can be automated to reduce design time. Only one type of material was used in this study. Different materials require further investigation to improve the moisture wicking issues that are systematic of perspiration. Only one participant with a specific level of amputation was recruited as a case study. Nevertheless, the aim of this work was to present a novel approach for rapidly manufacturing prosthetic liners with fully personalised designs in a cheap and fast method. A personalised liner that perfectly replicates the residual limb shape and with a specific design to embed sensors at the skin interface was developed. This begins with the scan of the residuum, followed by digital design and then manufacture through cryogenic CNC machining. Human testing, through this study has proven the feasibility of this new method. Future investigations will include a greater number of patients, each with a specific personalised liner. In this way, it will be possible to statistically characterise temperature and humidity changes at the level of residuum interface and confirm validity of the results on a larger sample of participants. The possibility to remotely monitor sensors within the liner will enable the patient condition to be monitored during prolonged activities.

Additional sensors could be included in the liner offering a solution for different needs to better characterise the main parameters affecting the prosthesis interface during clinical studies. The liner could be used as a data capture tool that transmits data to a closed-loop repository (*e.g.* motor-driven adjustable sockets for volume compensations [32]). It can also be used to monitor the conditions of the residuum and provide real-time feedback to the patient, or to collect or send data to prosthetists/clinicians via smart phone applications. Through this mechanism, the patients will be able to ensure that their liner and socket are suited to their needs and the occurrence of soft tissue damage can be significantly reduced impacting on quality of life.

## Methods

### Temperature and relative humidity characterization within the socket

Excessive heat and sweating within the socket are one of the most commonly reported complains from prosthetic users [2]. However, in the literature, only a few studies have been dedicated to the temperature assessment at the prosthetic socket interface [27–29], and only one to relative humidity [31]. Even if the characterization of the temperature and the relative humidity is of particular relevance to find new optimised prosthetic solutions, it has been notoriously complex because of the lack of appropriate measurement systems. Thermistor sensors, inserted within the socket (Fig. 1c), were typically used in previous studies. However, they are not wireless and could affect the prosthesis suspension during the test, thus resulting in misrepresented results and discomforts for the patient.

**Fig. 6:**
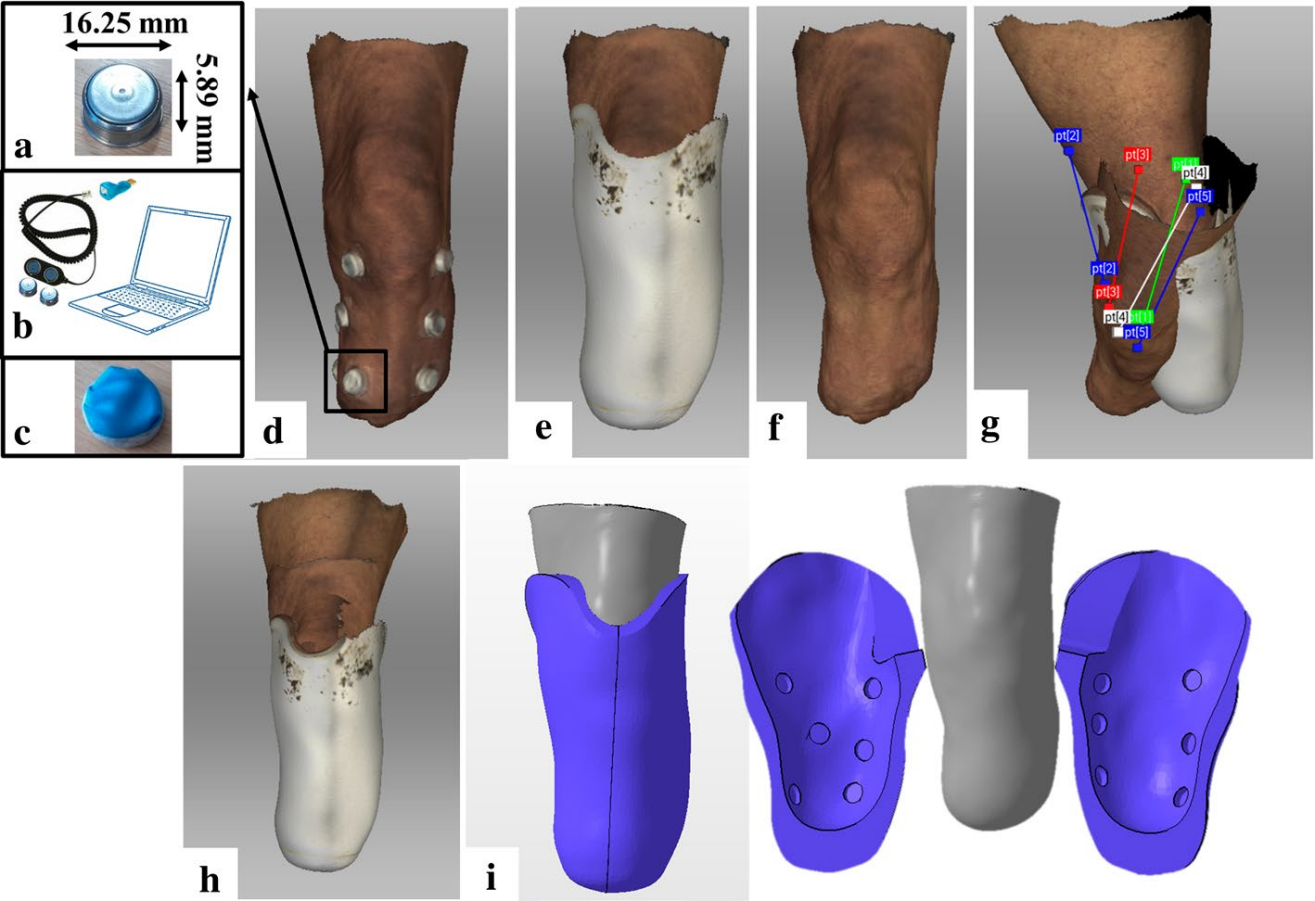
**a** Hygrochron Data Loggers (Type: DS1923-F5#) and **b** data acquisition system. **c** Hygrochron Data Loggers with the layer of breathable yet waterproof fabric; **d** Scan of residual limb with twelve Hygrochron Data Loggers positioned on; **e** Scan of the Pe-Lite liner; **f** Scan of the residual limb; **g** Alignment process; **h** Alignment result; **i** Final design of the new personalised liner with specific enclosures in the internal surface for the Hygrochron Data Loggers

Sensors able to collect both temperature and relative humidity data are now available (*e.g.* Hygrochron Data Loggers) and they can be used to better understand the thermal and humidity environment at the prosthetic socket interface. iButtons Data Loggers are small and wireless devices, characterised by a computer chip with a globally unique address enclosed in a stainless-steel case (diameter: 16.25-mm; thickness: 5.89-mm, Fig. 6a) (see [33] for more details). An iButton can include a read/write memory, real-time clocks and temperature and humidity data loggers. When data are recorded, a Blue Dot receptor cable and a 1-Wire USB adapter (*i.e.* the iButtons reader interface) is used to import data into a Laptop (see Fig. 6b). The OneWireViewer software is then used to analyse and save the recorded values.

Several studies reported their use for accurately measuring skin temperature and relative humidity in different clinical contexts without being invasive for patients [34, 35]. Twelve iButtons Data Loggers (Type: DS1923-F5# [33]) (Measurement System Ltd, Berkshire, UK) were selected for embedding into the personalised liner to measure temperature and relative humidity at the skin-prosthesis interface.

As the sensors were not waterproof, a layer of breathable, but waterproof, fabric was used to encase the sensors to prevent moisture ingress and damage (Fig. 6c). To improve the accuracy and detect any manufacturing defect, each iButton was calibrated using a climate chamber (ATT Climatic Chamber, Model DY110, ACS, Italy), before the data collection on the human amputee. The selected iButtons have an operating range equal to − 20 °C to + 85 °C for the temperature and 0–99% for the relative humidity [24]. During the calibration, the temperature ranged from 25 to 45 °C in steps of 5 °C, while the relative humidity from 20 to 95% in steps of 15%. The iButtons can be set to 8-bit (0.5 °C and 6% relative humidity) or 11-bit (0.0625 °C and 0.04% relative humidity) resolution. In this work, an 11-bit resolution was used. At the beginning of the calibration process, the iButtons were synchronised to collect data at a sampling time of 5 s for 15-min, after a 1-h waiting period which ensured a steady state condition of the climate chamber. This was done for each combination of temperature and relative humidity value (30 total values). Due to these settings, the sensors’ synchronisation had to be repeated every three temperature/relative humidity conditions before the logger memory was full. When the calibration process was completed, the collected data were exported through the sensor 1-Wire USB adapter, saved through the specific 1-Wire Software and exported to Excel. At each condition, the mean temperature and the mean relative humidity values were calculated over the 15-min interval. For both temperature and relative humidity, the mean values measured by the sensors were plotted against the values set in the climate chamber. Following this, the linear regression was obtained. This allowed for the correction of the measured sensor values. The improved accuracy (*i.e.* the mean of the differences between the values measured by the iButton and the climate chamber) and precision (*i.e.* the standard deviation of the differences between the values measured by the iButton and the climate chamber) were evaluated for each iButton as suggested by Shin et al. in 2017 [21].

### Participant and data acquisition

To design the new personalised liner, a unilateral below the knee (transtibial) amputee was recruited to participate in this institutional (Research Ethics Approval Committee for Health: REACH) review board-approved study (sex: male, age: 69 years, time post-amputation: 26 months). The participant’s socket system consisted of a Patellar Tendon Bearing carbon fiber-laminated socket with a Pe-Lite liner (made of medium-density polyethylene closed foam) and a SILOSHEATH™ prosthetic sheath (made of medical grade mineral oil gel and nylon outer material), suspended by a neoprene sleeve. Due to residual limb volume changes, the participant usually wore four socks to fit the prosthesis.

To properly evaluate the new liner outcomes, it was designed to fit the usual participant’s socket system. In fact, in previous studies, an objective comparison of the advantages and disadvantages of different liner solutions was often impossible, because they were affected by the use of different sockets and suspension systems in a single test session [6]. Using the patient’s current socket system, the effect of changes could only be due to the liner characteristics.

The 12 calibrated iButtons were positioned on the residual limb of the patient to find the most comfortable configuration (4 columns placed on the anterior-medial, anterior-lateral, posterior-medial and posterior-lateral locations, each of 3 equally spaced iButtons) (Fig. 6d). While the participant was standing on an elevated step in a standardised position, his residual limb was scanned to capture the sensors position. Then, the residual limb was scanned again with and without the amputee’s Pe-Lite liner (Fig. 6e and f). A handheld 3D Artec Eva structured light scanner (Artec Eva, Group, Luxembourg) was used to capture the residual limb volume and shape. This approach has been proven to be reliable and valid for assessing the shape and volume of an amputee’s residual limb [25, 36, 37].

### Design and manufacturing of the new personalised liner

The ad hoc sensorised liner has been designed and manufactured avoiding patient’s discomfort due to the iButtons thickness at the residual limb/prosthesis interface. The scans were post-processed using the Artec Studio 13 Professional software (Artec Studio, Artec Group, Luxemburg). As the scanner is able to collect texture/colour information, anatomic landmarks were used to properly align the 3D scans results with (Fig. 6e) and without (Fig. 1f) the amputee’s Pe-Lite liner, through the ‘*Complex Align’* function available in the software (Fig. 6g and h). This function allows for an advanced alignment within scans by selecting the desired landmark points on the pre-defined fixed scan and then the same points on the unregistered one. The scans were exported as solid CAD models (*.stl* files) and imported into the Autodesk Meshmixer software (Meshmixer, Autodesk Research, USA). Since the external surface of the Pe-Lite liner perfectly replicated the internal surface of the patient’s prosthetic socket, the residual limb model was subtracted from the Pe-Lite liner model by a Boolean operation. This allowed for the direct fit of the new liner with both the residual limb and the prosthetic socket. The internal surface of the new liner was smoothed, and its thickness increased $\sim$ 3-mm on the bony locations to increase the patient’s comfort (Fig. 6i). Specific enclosures have been added in the internal surface of the liner design to embed the temperature/humidity sensors at the interface with the skin (sensing systems mounting technique Fig. 1d), (Fig. 6i).

The manufacturing of the liner was performed using the cryogenic machining facility of the University of Bath: a Bridgeport VMC 610XP2 CNC Milling machine adapted into a functional cryogenic CNC machine process [38]. The process of cryogenic CNC machining is to freeze the specified material to below its glass transition temperature and, then, directly machine it using a standard CNC machine tool [16]. For ease of manufacture and patient use, the new liner design was divided into two parts (Fig. 6i). The final split liner design was imported into the Autodesk PowerMILL CAM software (PowerMILL, Autodesk Inc., USA) for the programming of the machining toolpaths. The material selected for the new liner was a type of neoprene closed foam due to its high impact attenuation properties and ease of freeing without excessive distortion or damage (Table 4). The neoprene foam workpiece was securely positioned and clamped in the machine fixture. Once the material workpiece had been located, the cryogenic spray jet system was activated to begin the freezing process. After the glass transition had been achieved (this is time and material dependent), the machining process was initialised. The machining process for a full liner takes approximately 4 h and depends on the complexity of the geometry.

**Table 4:** Neoprene foam material parameters

Specification	Value
Density	122 kg/m^3^
Temperature range	− 40: + 85 °C
Glass transition temperature (Tg)	− 45 °C
Time to achieve Tg	80 s
Young modulus at the Tg	1.3 GPa
Contraction factor	0.96%
Durometer value	15A–20A
Elongation at break ISO1798-7	> 180%
Tensile strength ISO1798-7	> 500 kPa
Water absorption, max change in weight	0.8%
Compression @25% deflection ASTMD1056-00	43 kPa

To securely match the two liner parts, a nylon sock was positioned and fixated around the external surface of the manufactured liner. Then, the new fully personalised liner weight was measured with a high precision measurement scale (KERN PFB 6000 2A, KERN & SOHN GmbH, Germany).

### Experimental protocol

**Fig. 7:**
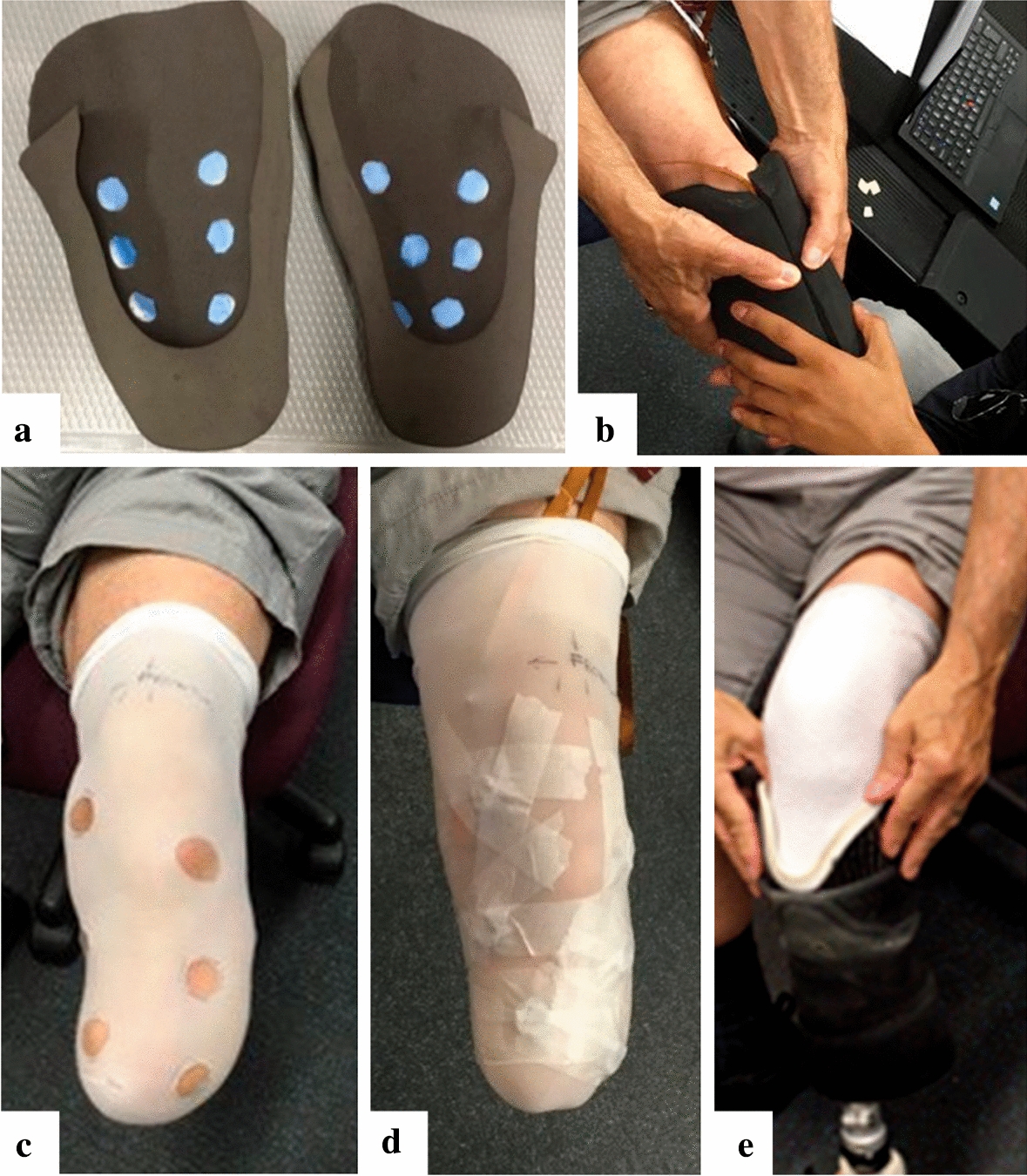
**a** The new *ad hoc* personalised liner with embedded sensors and **b** its fitting on the residual limb; **c** The modified SILOSHEATH™ prosthetic sheath with specific holes and **d** with the sensors in the same positions of the *ad hoc* personalised liner and **e** its fitting in the usual patient’s socket system

To confirm the effectiveness of the new approach, the transtibial amputee was asked to participate in a laboratory protocol to test the new personalised and sensorised liner during typical daily life activities and demonstrate the effectiveness of the new solution to characterise changes in skin temperature and humidity. In addition, a comparison between the new liner and the patient’s current prosthetic liner solution has been done in terms of measured data. This allowed for a more comprehensive analysis on the thermal properties of the proposed solution. The laboratory protocol involved two test sessions, each of 2-h and 10-min: 50-min seated resting period, 30-min of physical activity (walking at self-selected speed on the treadmill) and 50-min seated resting period post-exercise. A familiarisation period of 10-min was included before starting each session. During the first session, the participant was asked to wear the new personalised liner with the embedded temperature and relative humidity sensors (Fig. 7a, b). Then, the protocol was repeated in a separate session, where the same participant was asked to wear his liner system (four layers of socks, one Pe-Lite Liner and the SILOSHEATH™ prosthetic sheath). To reduce the discomfort of the iButtons at the prosthesis/skin interface, a new SILOSHEATH™ prosthetic sheath was used for the second session tests. Using a hollow punch tool, specific holes were made to accommodate the iButton sensors in the same positions as on the fully personalised liner (Fig. 7c–e), adopting the sensing systems mounting technique by integrating sensors on the liner wall (Fig. 1a). To monitor the room conditions, before starting the human tests, the temperature and the relative humidity were recorded by the iButton sensors positioned on a table in a central location of the laboratory.

In addition, images of the residual limb were captured by a FLIR ONE thermo-camera connected to a Smartphone. Due to the Multi-Spectral Dynamic Technology (MSX) [39], the thermo-camera fuses standard photos with thermal images. This allowed for an additional qualitative characterization of the skin temperature distribution after wearing the socket with the two liner solutions for the duration of the test.

Since the liner comfort is a subjective experience, best described by the limb user, the Socket Comfort Score (SCS) [40] and a modified Prosthesis Evaluation Questionnaire (PEQ) [41] were proposed to the patient to assess the comfort and the acceptance of the new personalised liner. Both these tools are based on the subjective symptoms of the patient. While the SCS is based on a 0–10 scale and it is similar to the pain measurement systems, the PEQ includes different domains. It was developed specifically to provide functional outcome measures in prosthetics that are more tuned to prosthesis-related changes in quality of life. PEQ results are expressed in percentages (higher scores implicate high comfort).

### Data processing

The data collected during human tests were exported through the sensor 1-Wire USB adapter system and saved in a *CSV* file through the specific 1-Wire Software. This was then imported into MATLAB R2018a: for each sensor, the measured temperature and relative humidity data were corrected by the individual correction formula based on the linear regression and calculated during the calibration process. The mean temperature and relative humidity curves were evaluated for each test session.

Different 1-min stages of the tests were identified during the different phases of the data collection and for each of them the average and standard deviation were calculated both for temperature and humidity, for the mean curve and for each of the iButton sensors. A t-test for paired data was used in IBM SPSS Statistics to evaluate possible statistically significant differences between the new personalised liner and the Pe-Lite liner system in terms of temperature and relative humidity for each of the different stages of the tests, using the averaged data of each of the 12 sensors.

## Conclusions

This paper presents a case study that describes the design and development of a new fully personalised liner solution with embedded temperature and humidity sensors via a semi-automated process for a below knee amputee. Monitoring the main parameters influencing this interface (*e.g.* temperature and humidity) is fundamental to design optimised prosthetic socket solutions and assess the residuum environment. However, proper fit between the residual limb and the sensorised interface is crucial for the comfort and the performance of the patient’s prostheses. The direct manufacturing approach presented in this paper begins with the 3D scan of the residuum of the amputee. The liner is then designed accordingly with the residuum shape characteristics which can include a variety of sensors to be embedded in liner. The cryogenic CNC machining approach allows for the manufacture of low-cost personalised liner solutions within a few hours. The proposed liner has shown to be a potential solution for prosthesis fitting and sensorised interface. It is light and quick to wear compared with other solutions and it can maintain lower temperatures at the residuum interface (on average 4% lower compared to the conventional solution across different types of activities).

Future tests including a larger sample of participants are needed to assess the liner under different fatigue conditions. Different sensors can be embedded to measure different parameters providing real-time feedback for the patient and the clinician.

### Supplementary information

**Additional file 1**. Temperature and relative humidity mean values of the residual limb skin for each sensor.

## Data Availability

The codes and datasets of this study are available from the corresponding author upon reasonable request.
