# Correction: A personalised prosthetic liner with embedded sensor technology: a case study

**DOI:** 10.1186/s12938-023-01154-3

**Published:** 2023-09-21

**Authors:** Linda Paternò, Vimal Dhokia, Arianna Menciassi, James Bilzon, Elena Seminati

**Affiliations:** 1 Department of Mechanical Engineering, University of Bath, Bath, UK; 2 The BioRobotics Institute, Scuola Superiore Sant’Anna, Pisa, Italy; 3 Department for Health, University of Bath, Bath, UK; 4 CAMERA Centre, University of Bath, Bath, UK

**Correction: BioMed Eng OnLine (2020) 19:71.** **https://doi.org/10.1186/s12938-020-00814-y**

Following publication of the original article [1], in this article the “Method” and “Conclusion” section needs to be interchanged and the same has been updated.

The original article has been corrected.
