# Supplementary material for: A personalised prosthetic liner with embedded sensor technology: a case study
Source: Biomed Eng Online. 2020 Sep 14;19:71. doi: 10.1186/s12938-020-00814-y (PMC7491094; doi:10.1186/s12938-020-00814-y)
Supplement: Supplementary file 1 — Additional file 1. Temperature and relative humidity mean values of the residual limb skin for each sensor. [file 12938_2020_814_MOESM1_ESM.docx]

**Table 1.** Temperature and relative humidity mean values of the residual limb skin for each sensor. Data were averaged on the initial/ final 1-minute recorded data of each task.

| **Sensor** |  | **New Liner** | | **Pe-Lite Liner System** | | |
| --- | --- | --- | --- | --- | --- | --- |
|  |  | **T_mean_ ± std [°C]** | **RH_mean_ ± std [%]** | | **T_mean_ ± std [°C]** | **RH_mean_ ± std [%]** |
| 1 | *Start 1^st^ resting period* | 31.44 ± 0.02 | 68.83 ± 0.47 | | 32.97 ± 0.01 | 75.93 ± 0.42 |
|  | *End 1^st^ resting period* | 31.58 ± 0.01 | 78.21 ± 0.51 | | 34.14 ± 0.05 | 79.79 ± 0.99 |
|  | *End physical activity* | 34.24 ± 0.02 | 80.25 ±0.44 | | 35.87 ± 0.02 | 82.97 ± 0.46 |
|  | *End 2^nd^ resting period* | 33.95 ± 0.01 | 84.95 ± 0.41 | | 35.03 ± 0.04 | 87.04 ± 0.42 |
| 2 | *Start 1^st^ resting period* | 31.04 ± 0.01 | 68.40 ± 0.29 | | 33.06 ± 0.03 | 71.98 ± 0.28 |
|  | *End 1^st^ resting period* | 30.85 ± 0.01 | 77.74 ± 0.56 | | 34.06 ± 0.05 | 74.35 ± 0.41 |
|  | *End physical activity* | 33.71 ± 0.04 | 81.41 ± 0.40 | | 36.03 ± 0.01 | 78.81 ± 0.34 |
|  | *End 2^nd^ resting period* | 33.54 ± 0.01 | 83.96 ± 0.35 | | 35.34 ± 0.01 | 80.94 ± 0.37 |
| 3 | *Start 1^st^ resting period* | 29.81 ± 0.01 | 70.66 ± 0.25 | | 32.56 ± 0.01 | 76.77 ± 0.43 |
|  | *End 1^st^ resting period* | 29.37 ± 0.01 | 80.14 ± 0.38 | | 33.10 ± 0.03 | 84.70 ± 0.44 |
|  | *End physical activity* | 33.94 ± 0.04 | 82.08 ± 0.39 | | 35.73 ± 0.02 | 85.64 ± 0.57 |
|  | *End 2^nd^ resting period* | 32.91 ± 0.03 | 85.34 ± 0.51 | | 34.41 ± 0.03 | 89.12 ± 0.24 |
| 4 | *Start 1^st^ resting period* | 31.74 ± 0.03 | 67.87 ± 0.27 | | 33.07 ± 0.01 | 72.82 ± 0.35 |
|  | *End 1^st^ resting period* | 31.76 ± 0.01 | 78.25 ± 0.47 | | 33.85 ±0.03 | 81.87 ± 0.43 |
|  | *End physical activity* | 34.52 ± 0.03 | 81.91 ± 0.31 | | 36.11 ± 0.03 | 83.50 ± 0.49 |
|  | *End 2^nd^ resting period* | 33.94 ± 0.01 | 84.18 ± 0.26 | | 35.50 ± 0.01 | 86.62 ± 0.38 |
| 5 | *Start 1^st^ resting period* | 31.86 ± 0.01 | 67.87 ± 0.27 | | 33.19 ± 0.02 | 72.82 ± 0.35 |
|  | *End 1^st^ resting period* | 31.49 ± 0.01 | 78.25 ± 0.47 | | 33.60 ± 0.02 | 81.87 ± 0.43 |
|  | *End physical activity* | 35.09 ± 0.03 | 81.91 ± 0.31 | | 36.11 ± 0.01 | 83.50 ± 0.43 |
|  | *End 2^nd^ resting period* | 34.38 ± 0.03 | 84.18 ± 0.26 | | 35.23 ± 0.01 | 86.62 ± 0.38 |
| 6 | *Start 1^st^ resting period* | 30.62 ± 0.01 | 68.80 ± 0.67 | | 32.13 ± 0.01 | 71.84 ± 0.68 |
|  | *End 1^st^ resting period* | 30.19 ± 0.01 | 77.70 ± 0.45 | | 33.05 ± 0.02 | 78.57 ± 0.35 |
|  | *End physical activity* | 35.53 ± 0.03 | 79.27 ± 0.39 | | 35.51 ± 0.03 | 81.34 ± 0.47 |
|  | *End 2^nd^ resting period* | 33.33 ± 0.02 | 84.56 ± 0.27 | | 33.76 ± 0.02 | 84.30 ± 0.31 |
| 7 | *Start 1^st^ resting period* | 32.27 ± 0.01 | 69.64 ±0.45 | | 32.89 ± 0.01 | 68.52 ± 0.49 |
|  | *End 1^st^ resting period* | 32.52 ± 0.01 | 80.32 ± 0.38 | | 33.51 ±0.02 | 75.07 ± 0.38 |
|  | *End physical activity* | 34.65 ±0.04 | 83.37 ± 0.39 | | 35.33 ± 0.02 | 77.24 ± 0.44 |
|  | *End 2^nd^ resting period* | 34.64 ± 0.01 | 85.91 ± 0.32 | | 35.02 ± 0.01 | 78.87 ± 0.39 |
| 8 | *Start 1^st^ resting period* | 31.92 ± 0.01 | 67.72 ± 0.43 | | 33.05 ± 0.01 | 70.56 ± 0.51 |
|  | *End 1^st^ resting period* | 31.65 ± 0.03 | 77.63 ± 0.40 | | 33.72 ± 0.03 | 77.94 ± 0.27 |
|  | *End physical activity* | 34.89 ± 0.04 | 80.99 ± 0.37 | | 35.73 ± 0.01 | 80.16 ± 0.35 |
|  | *End 2^nd^ resting period* | 34.03 ± 0.02 | 84.21 ± 0.39 | | 34.92 ± 0.01 | 82.65 ± 0.31 |
| 9 | *Start 1^st^ resting period* | 31.82 ± 0.01 | 67.77 ± 0.59 | | 33.32 ± 0.01 | 75.83 ± 0.67 |
|  | *End 1^st^ resting period* | 31.01 ± 0.01 | 76.97 ± 0.19 | | 33.58 ± 0.05 | 81.35 ± 0.37 |
|  | *End physical activity* | 35.51 ± 0.03 | 78.18 ± 0.58 | | 35.57 ± 0.01 | 83.52 ± 0.60 |
|  | *End 2^nd^ resting period* | 33.83 ± 0.02 | 83.73 ± 0.49 | | 34.52 ± 0.02 | 85.12 ± 0.40 |
| 10 | *Start 1^st^ resting period* | 32.70 ± 0.03 | 66.47 ± 0.23 | | 32.85 ± 0.01 | 67.59 ± 0.18 |
|  | *End 1^st^ resting period* | 32.81 ± 0.02 | 75.80 ± 0.21 | | 33.30 ± 0.02 | 76.57 ± 0.46 |
|  | *End physical activity* | 34.74 ± 0.04 | 78.47 ± 0.37 | | 35.41 ± 0.02 | 79.42 ± 0.26 |
|  | *End 2^nd^ resting period* | 35.10 ± 0.01 | 82.13 ± 0.24 | | 34.86 ± 0.02 | 81.79 ± 0.46 |
| 11 | *Start 1^st^ resting period* | 31.66 ± 0.01 | 67.71 ± 0.25 | | 33.18 ± 0.02 | 68.46 ± 0.35 |
|  | *End 1^st^ resting period* | 31.29 ± 0.01 | 76.61 ± 0.28 | | 33.40 ± 0.03 | 76.91 ± 0.55 |
|  | *End physical activity* | 34.41 ± 0.04 | 79.13 ± 0.43 | | 35.81 ± 0.03 | 78.05 ± 0.47 |
|  | *End 2^nd^ resting period* | 34.16 ± 0.01 | 82.75 ± 0.26 | | 34.77 ± 0.02 | 80.70 ± 0.34 |
| 12 | *Start 1^st^ resting period* | 30.12 ± 0.01 | 67.856 ± 0.31 | | 33.01 ± 0.02 | 67.80 ± 0.38 |
|  | *End 1^st^ resting period* | 29.87 ± 0.01 | 76.75 ± 0.28 | | 32.98 ± 0.02 | 75.06 ± 0.44 |
|  | *End physical activity* | 34.18 ± 0.04 | 78.87 ± 0.38 | | 35.34 ± 0.01 | 76.76 ± 0.66 |
|  | *End 2^nd^ resting period* | 33.12 ± 0.01 | 82.92 ± 0.29 | | 34.24 ± 0.01 | 80.26 ± 0.48 |
